# Severe Airway Obstruction Caused by Esophageal Bezoar with Coca-Cola and Creon (Pancrelipase) in a Patient with Underlying Achalasia: A Comprehensive Case Report

**DOI:** 10.1155/2024/2081040

**Published:** 2024-07-27

**Authors:** Kinnera Sahithi Urlapu, Nikhitha Mantri, Harish Patel, Priscilla Lajara Hallal, Sridhar Chilimuri, Gilda Diaz-Fuentes

**Affiliations:** ^1^ Division of Pulmonary and Critical Care Department of Medicine Bronx Care Health System Affiliated with Icahn School of Medicine at Mount Sinai, New York, Bronx, USA; ^2^ Division of Gastroenterology Department of Medicine Bronx Care Health System Affiliated with Icahn School of Medicine at Mount Sinai, New York, Bronx, USA; ^3^ Department of Medicine Bronx Care Health System Affiliated with Icahn School of Medicine at Mount Sinai, New York, Bronx, USA

## Abstract

**Introduction:**

The occurrence of acute respiratory failure as a result of esophageal bezoars is a rare phenomenon. We present a patient who failed initial endoscopic intervention. Successful resolution was achieved through a novel approach involving a combination of Creon and Coca-Cola. Subsequently, the patient was diagnosed with achalasia, a condition that potentially contributed to the formation of the esophageal bezoar. *Case Description*. An 82-year-old man presented with respiratory distress, necessitating endotracheal intubation for airway protection. A chest computed tomogram (CT) showed pneumonia and a distended esophagus compressing the trachea, raising the possibility of an esophageal food bolus. Endoscopy revealed a severely dilated esophagus containing a significant amount of food and a phytobezoar in the lower esophagus. He failed various endoscopic techniques to remove the obstruction. Given the patient's poor surgical candidacy, he was started in a thrice-daily regimen of Creon dissolved in 165 mL of Coca-Cola, over a 4-day period. A subsequent endoscopy revealed no discernible evidence of food or bezoar. The patient was weaned from mechanical ventilation. A high-resolution esophageal manometry identified type 1 achalasia.

**Conclusion:**

Esophageal food impaction leading to respiratory failure is rare. Endoscopy remains the mainstay approach. Surgical interventions carry significant risks. This case emphasizes the potential for noninvasive management in patients with esophageal bezoars and also underscores the significance of contemplating esophageal pathologies when addressing cases of respiratory failure. The use of Coca-Cola and Creon emerges as a safe, effective, and cost-efficient treatment, providing a feasible option when endoscopy proves unsuccessful before considering more aggressive interventions.

## 1. Introduction

Esophageal bezoars are a rare occurrence, characterized by the formation of a mass or foreign body within the esophagus. While some cases may be asymptomatic, others typically present with gastrointestinal (GI) symptoms like dysphagia, odynophagia, weight loss, regurgitation, etc. Remarkably, esophageal bezoars can also manifest atypically, leading to symptoms like respiratory distress, necessitating urgent measures like airway stabilization and mechanical ventilation in severe cases.

The successful management of esophageal bezoars involves a nuanced approach, considering factors such as the size, location, and composition of the bezoar, complications associated as well as underlying comorbid conditions of the patient. Medical interventions or surgical removal may be employed based on these considerations. Furthermore, it is crucial to recognize that patients with bezoars may have underlying medical conditions contributing to their formation. Identifying and addressing these underlying illnesses promptly are vital to prevent recurrence and mitigate the progression of the associated diseases. In essence, the management of bezoars requires a comprehensive understanding of their diverse presentations. Tailoring interventions to individual cases, along with addressing underlying medical conditions, is essential for effective and holistic patient care.

We hereby present a case of a patient experiencing respiratory distress attributed to an esophageal bezoar that proved resistant to conventional endoscopic management. Successful resolution was achieved through a combination of Creon and Coca-Cola. Subsequent investigation into the cause of the bezoar unveiled an underlying diagnosis of achalasia.

## 2. Case Report

An 82-year-old male with a medical history of prediabetes, hypertension, hypothyroidism, benign prostatic hyperplasia, vertigo, and anxiety presented to the emergency room with complaints of nausea, vomiting, dizziness, and respiratory distress. He denied chest pain, abdominal pain, palpitations, cough, fever, headache, GI bleeding, or leg swelling. He had no significant surgical history and drug allergies and reported no history of smoking, alcohol, or drug use. His medications included clonazepam, amlodipine, levothyroxine, and tamsulosin.

On examination, the patient displayed significant respiratory distress, including choking, gagging, and stridor. Initial vital signs indicated oxygen saturation in the 90s on ambient air, with a tachycardic heart rate of 120 beats per minute. Physical examination revealed coarse breath sounds on lung auscultation. Neurological, cardiovascular, and gastrointestinal examinations were unremarkable. Due to severe respiratory distress, the patient required intubation and mechanical ventilation.

**Figure 1:**
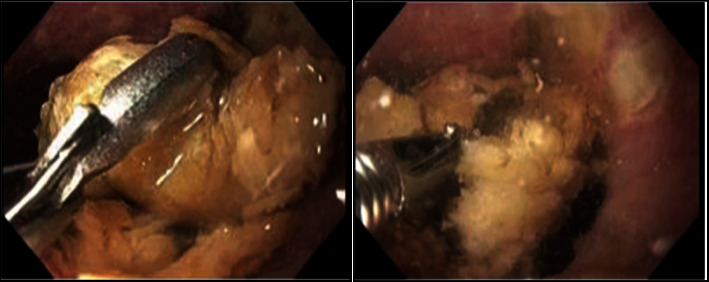
Initial endoscopic images capturing the lower third of the esophagus, displaying the presence of an impacted bezoar. Visible attempts to break down the bezoar using rat tooth forceps are documented.

An urgent direct laryngoscopy revealed no abnormalities explaining the symptoms. Chest CT was performed to evaluate the etiology of respiratory distress, ruling out pulmonary embolism but revealing near-complete atelectasis of the left lower lobe and a distended esophagus with a suspected ingested bolus above the carina. A subsequent esophagogastroduodenoscopy (EGD) disclosed a severely dilated esophagus with food throughout and a bezoar in the lower esophagus (Figure 1). Attempts to remove the bezoar using various instruments like raptor forceps, Roth net, and talon grasping forceps were unsuccessful. A bronchoscopy was performed which showed purulent secretions and food debris in the bilateral lung lobes.

**Figure 2:**
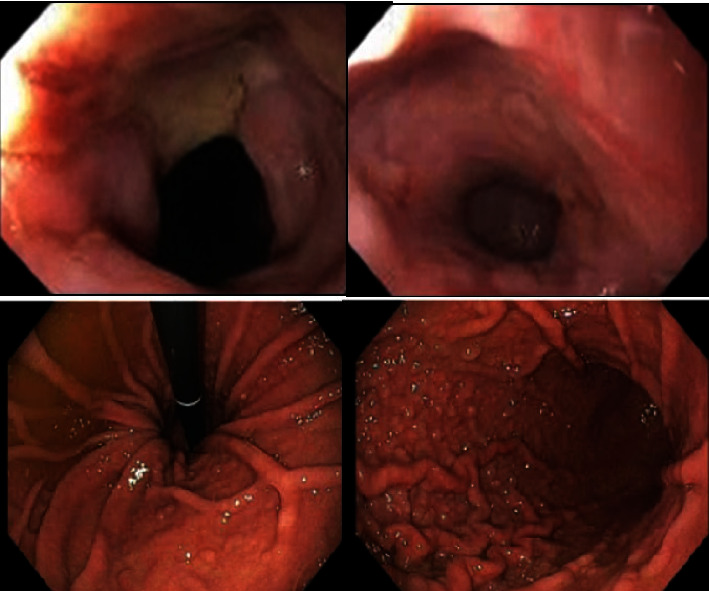
Endoscopic evaluation conducted day five post-Coca-Cola-Creon therapy revealed clearing of bezoar and esophagitis with circumferential ulcer in lower with hiatal hernia.

Considering the high morbidity and mortality associated with surgery in this elderly patient, surgical intervention was deferred. Since endoscopic management proved ineffective, a decision was made to pursue medical management. We planned for dissolution of bezoars using an oro-gastric tube for direct purging of carbonated fluids infused with pancreatic enzymes. The oro-gastric tube was inserted blindly to a depth of 20 centimeters from the incisors, with the objective of lavaging from the upper esophagus to fully submerge the bezoar. In our patient, endotracheal intubation mitigated the risk of tracheal aspiration. In patient with no prior endotracheal intubation, direct laryngoscopy can be performed to ensure the esophageal intubation of the oro-gastric tube. The patient received a combination of Coca-Cola and Creon via an oro-gastric tube, with 1 can (355 mL) of Coca-Cola and 4 capsules of Creon administered four times a day for 4 days. A repeat EGD on day 5 revealed complete resolution of the bezoar but identified esophagitis with circumferential ulceration and a 5-cm hiatal hernia, with no residual food in the gastric body (Figure 2). Biopsies of the lower and middle esophagus were performed to evaluate eosinophilic esophagitis. Histopathological examination revealed squamous mucosa with reactive changes, and there was an absence of eosinophilia, thereby ruling out eosinophilic esophagitis.

The patient clinically improved, successfully weaning off mechanical ventilation on day 6 of hospitalization. High-resolution esophageal manometry was performed with endoscopic placement of the manometry catheter, which revealed the integrated relaxation pressure (IRP) of 36 mm of Hg (normal of 20 mm of Hg) with ineffective esophageal motility in the 100% of the swallows and confirmed the diagnosis of type I achalasia. Upon discharge, the patient received proton pump inhibitors and was scheduled for outpatient follow-up with gastroenterology for further management of achalasia and esophagitis.

## 3. Discussion

Bezoars are aggregations of indigestible foreign materials within the gastrointestinal tract. They can be categorized based on their composition, with phytobezoars being the most prevalent form, resulting from undigested food materials. Trichobezoars, formed by ingested hair, lactobezoars observed in milk-fed neonates, and pharmacobezoars originating from undigested medicines represent other classifications [1, 2].

The clinical presentation of bezoars is variable, encompassing a spectrum from asymptomatic cases to symptomatic manifestations such as abdominal pain, distention, dysphagia, anemia, nausea, vomiting, weight loss, gastrointestinal bleeding, and even bowel obstruction [2–4].

Extraintestinal symptoms are uncommon in the clinical presentation of bezoars. Respiratory distress caused by esophageal bezoars is a rare phenomenon, albeit documented in existing literature. The reported mechanisms underlying this phenomenon involve several factors. One such factor is the restriction in lung volumes, where the presence of large esophageal bezoars may impede the normal expansion of the lungs, leading to respiratory difficulties [5]. Additionally, aspiration of esophageal contents, such as the bezoar material, into the airways can contribute to respiratory distress [5, 6]. Moreover, the challenge in weaning patients off ventilator support may arise due to the obstructive nature of esophageal bezoars, making the process of transitioning from mechanical ventilation more difficult [7]. Another documented mechanism involves the occurrence of respiratory distress due to bezoar obstruction lodged in the hypopharynx [8]. These multifaceted mechanisms collectively contribute to the respiratory complications associated with esophageal bezoars.

**Figure 3:**
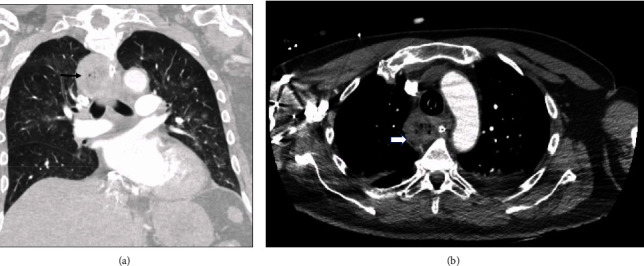
(a) Coronal view of CT chest with contrast showing a large impacted food bolus just above the carina (black arrow). (b) Axial view of CT chest with contrast showing food bolus with air (white arrow). A nasogastric tube can be seen traversing the food bolus.

**Figure 4:**
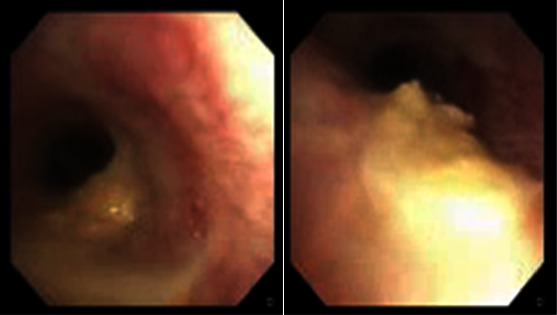
Fiberoptic bronchoscopy images depicting the presence of purulent secretions and food particles observed in the left lower lobe.

In our case, the esophageal bezoar led to this rare complication, manifesting as respiratory distress. The extrinsic compression on the trachea resulted from the physical presence of the esophageal bezoar, causing pressure on the airway (Figures 3(a) and 3(b)). Simultaneously, the aspiration of bezoar contents into the tracheobronchial tree added another layer of complexity. Aspiration involved the inhalation of foreign material into the respiratory passages (Figure 4), potentially leading to respiratory compromise.

This dual mechanism, involving both extrinsic compression and aspiration, represents an uncommon and noteworthy manifestation of esophageal bezoar-related respiratory complications in our specific case. In the context of aspiration pneumonia, respiratory decompensation may ensue. The development of a bezoar is typically gradual, but aspiration represents a critical tipping point that can precipitate respiratory decompensation. Understanding these intricate factors is crucial for effective management and underscores the need for individualized approaches in such rare presentations. In rare instances, like in our patient, the initial symptoms were misleading, suggesting a respiratory origin. It is paramount to consider these potential differentials, as an early and accurate diagnosis, coupled with timely intervention, can significantly reduce morbidity and mortality in such cases.

Another intriguing aspect of this case is the management of esophageal bezoar in our patient. While endoscopic management continues to remain the primary diagnostic and therapeutic method for bezoars, alternative noninvasive methods have been used previously either alone or in conjunction with endoscopic therapy for successful management of bezoars. Compounds like cellulase, cysteine, sodium bicarbonate, ginger-ale, and papain are among the few that have historically been employed in the dissolution of bezoars [9–13]. Gupta et al. were among the early researchers to document the successful breakdown of esophageal bezoars through the application of pancreatic enzymes [14]. Ladas et al. reported the initial case of utilizing Coca-Cola for the dissolution of a stomach phytobezoar [15]. Ota et al. reported a study on the dissolution of phytobezoars through direct endoscopic injection of Coca-Cola into the bezoar, a technique that can be employed as an additional method for delivering Coca-Cola directly to the bezoar [16]. Additionally, Yakub et al. recorded the first instance of achieving esophageal bezoar resolution through a combination of Coca-Cola and pancreatic enzymes [17]. Subsequently, multiple studies have documented the effective dissolution of bezoars utilizing Coca-Cola, pancreatic enzymes, a combination of both, or in conjunction with endoscopic therapy [13, 15, 18–22].

The precise mechanisms driving the efficacy of Coca-Cola and Creon therapy remain incompletely understood, though certain theories have been proposed. Considering the significance of acid in fiber digestion, Coca-Cola, containing carbonic and phosphoric acid with a pH of 2.3–2.6, akin to the pH range of 1-2 in normal gastric secretions, is theorized to acidify the bezoar contents. This acidity, in combination with the liberation of carbon dioxide bubbles, is suggested to contribute to the disintegration of phytobezoars [23–25]. Furthermore, Coca-Cola has been demonstrated to reduce the tone of the lower esophageal sphincter (LES) and enhance the frequency of transient LES relaxation [26]. This effect could potentially play a role in facilitating the movement of the esophageal bezoar into the stomach. One of the mechanisms of formation of bezoars is believed to be linked to the coagulation of casein at a low pH. Highlighting the significance, it has been identified that pancreatic enzymes serve as inhibitors of the coagulation of this substance, facilitating its liquefaction in vitro [14, 27]. This characteristic is likely the basis for considering their use in bezoar dissolution.

In our case, the successful dissolution of the bezoar using a combination of Coca-Cola and Creon underscores the significance of employing a noninvasive and cost-efficient approach. This method, which leverages the acidic properties of Coca-Cola and the enzymatic action of Creon, provides a feasible alternative to more invasive interventions, potentially reducing the associated morbidity and mortality. The impact of Coca-Cola on the LES may have contributed to facilitating the movement of the esophageal bezoar into the stomach, thus potentially alleviating the compressive effect of the esophagus on the trachea in our case. The utilization of this approach highlights the importance of considering alternative methods in the management of esophageal bezoars, particularly in such presentations where endoscopic techniques prove ineffective.

Table 1 presents the summary of studies depicting treatment of esophageal and gastric bezoars using Coca-Cola and/or pancreatic enzymes, either independently or in conjunction with endoscopic therapy.

Another noteworthy finding from our study is the identification of underlying achalasia in our patient. Various risk factors contribute to bezoar formation, including gastric dysmotility conditions (such as gastroparesis, previous gastric surgeries, and vagotomy), anatomical abnormalities (such as gastric diverticula, and pyloric stenosis), psychiatric disorders, and diabetes, among others [4, 20, 32–34].

Esophageal motility disorders, identified as a risk factor for esophageal bezoar formation, include conditions such as achalasia, myasthenia gravis, Guillain–Barré syndrome, and diffuse esophageal spasm. [28, 30, 31]. The mechanisms facilitating esophageal bezoar formation in these patients include abnormal lower esophageal sphincter (LES) relaxation, abnormal esophageal peristalsis or aperistalsis, abnormal gastric emptying, and various other factors [28]. Symptoms in these patients can vary, commonly presenting with GI symptoms such as nausea, vomiting, dysphagia, odynophagia, and weight loss, or they can be asymptomatic. In summary, the revelation of underlying achalasia in our patient highlights the paramount importance of diagnosing associated conditions in clinical presentations of esophageal bezoars. The timely recognition and diagnosis of underlying conditions can play a pivotal role in guiding appropriate interventions and enhancing outcomes for patients.

## 4. Conclusion

In conclusion, our case highlights the complexities involved in managing life-threatening respiratory failure caused by upper airway obstruction due to an esophageal bezoar. Despite initial challenges with standard endoscopic approaches, the use of Coca-Cola and Creon therapy not only successfully dissolved the bezoar but also led to the patient's liberation from mechanical ventilation.

When faced with esophageal bezoar-related upper airway obstruction symptoms, albeit rare, prompt consideration and evaluation of underlying esophageal pathology are crucial. Early airway stabilization, coupled with appropriate diagnostics, is imperative, and the decision to use carbonated drinks or endoscopic techniques should be individualized. Our experience highlights the significance of adaptability and innovation in addressing challenging clinical scenarios, demonstrating the potential of unconventional therapies for favorable outcomes.

## Figures and Tables

**Table 1:** Comprehensive literature review on esophageal bezoars in patients with underlying achalasia.

Serial number	Type of Bezoar	Treatment of bezoar	Reference number
1	Phytobezoar	Endoscopic removal	[28]
2	Pharmacobezoar	Endoscopic removal	[29]
3	Phyto-pharmacobezoar	Endoscopic removal	[30]
4	Phytobezoar	Endoscopic removal	[31]
5	Phytobezoar	Coca-Cola and Creon	Our case
